# Neuronal basis and diverse mechanisms of pathogen avoidance in *Caenorhabditis elegans*

**DOI:** 10.3389/fimmu.2024.1353747

**Published:** 2024-05-01

**Authors:** Ming Lei, Yanheng Tan, Haijun Tu, Weihong Tan

**Affiliations:** ^1^Academy of Medical Engineering and Translational Medicine (AMT), Tianjin University, Tianjin, China; ^2^State Key Laboratory of Chemo/Biosensing and Chemometrics, College of Biology, Hunan University, Changsha, Hunan, China; ^3^The Key Laboratory of Zhejiang Province for Aptamers and Theranostics, Zhejiang Cancer Hospital, Hangzhou Institute of Medicine (HIM), Chinese Academy of Sciences, Hangzhou, Zhejiang, China

**Keywords:** pathogen avoidance, aerotaxis, metabolites, neural circuit, reactive oxygen species, innate immunity

## Abstract

Pathogen avoidance behaviour has been observed across animal taxa as a vital host-microbe interaction mechanism. The nematode *Caenorhabditis elegans* has evolved multiple diverse mechanisms for pathogen avoidance under natural selection pressure. We summarise the current knowledge of the stimuli that trigger pathogen avoidance, including alterations in aerotaxis, intestinal bloating, and metabolites. We then survey the neural circuits involved in pathogen avoidance, transgenerational epigenetic inheritance of pathogen avoidance, signalling crosstalk between pathogen avoidance and innate immunity, and *C. elegans* avoidance of non-*Pseudomonas* bacteria. In this review, we highlight the latest advances in understanding host-microbe interactions and the gut-brain axis.

## 1 Introduction

Pathogen avoidance is a pivotal behavioural adaptation observed in diverse animal species within natural ecosystems. It serves as a mechanism to distinguish safe food sources from potential threats posed by harmful bacteria and lethal pathogens. This adaptive response, an integral component of the behavioural immune system, plays a fundamental role in individual survival, thereby influencing the persistence of populations within their ecological niches (1). Various species, including mice (2), bonobos (pygmy chimpanzees) (3), chimpanzees (4), and humans (5), demonstrate the capacity for pathogen avoidance.

*Caenorhabditis elegans* (*C. elegans*), a nematode that lives in microbe-rich soils (6, 7), is also capable of avoiding pathogens. Given its habitat, *C. elegans* inevitably encounters pathogenic bacteria, prompting the development of sophisticated avoidance mechanisms via natural selection. One such pathogen is the opportunistic human pathogen *Pseudomonas aeruginosa* (PA14) (7, 8). *C. elegans* is initially attracted to PA14, but over time, *C. elegans* avoids PA14 by aversive learning (9). Such behavioural avoidance has emerged as a valuable model for unravelling the intricacies of pathogen avoidance mechanisms. In this review, we will first discuss various stimuli that promote pathogen avoidance. Next, we will describe the neural circuitry and the underlying neural and molecular mechanisms that regulate pathogen avoidance. We will also cover the recently discovered role of small non-coding RNAs in transgenerational inheritance of pathogen avoidance and the role of small non-coding RNAs. Finally, we will conclude this review by discussing the crosstalk between innate immunity and pathogen avoidance, as well as the *C. elegans* avoidance of pathogens other than PA14. This review aims to comprehensively summarise these scientific research achievements and provide a systematic overview of pathogen avoidance behaviours in *C. elegans*.

## 2 Stimuli that trigger pathogen avoidance and the underlying molecular mechanisms

### 2.1 Aerotaxis

Aerotaxis refers to the behavioural movement of animals in response to oxygen concentrations in the environment. *C. elegans* migrates towards regions with 5–12% oxygen levels but avoids regions with higher (>12%) and lower (<2%) oxygen concentrations (10). The haem domain of GCY-35, a specific soluble guanylate cyclase homologue, binds to molecular oxygen. GCY-35 activity is regulated by molecular oxygen, which subsequently produces 3’,5’-cyclic guanosine monophosphate (cGMP) that acts as a second messenger and is implicated in oxygen sensation (10). TAX-4, a cyclin nucleotide-gated channel, is activated by GCY-35 through cGMP in URX, AQR, and PQR sensory neurons to promote the aggregation of animals on a bacterial lawn and their accumulation on the thickest part of the bacterial lawn (known as bordering behaviour). The processes of aggregation and boarding mediated by GCY-35 and TAX-4 are antagonised by the activity of the neuropeptide receptor NPR-1 (11, 12). Hyperoxia avoidance regulated by NPR activity requires the neurotransmitter serotonin in ADF sensory neurons. The neuronal TGF-beta homologue DAF-7 inhibits serotonin synthesis in ADF neurons, thereby regulating hyperoxia avoidance (10). Interestingly, it has been determined that alginate biosynthesis in mucoid *P. aeruginosa* suppresses NPR-1-mediated pathogen avoidance behaviour (12). GCY-35 and GCY-36 expressed in the head and tail neurons modifies *C. elegans* movement, promoting reversal and turning when oxygen levels increase. Hyperoxia avoidance is also controlled by the TRP-related channel subunits OCR-2 and OSM-9 and the transmembrane protein ODR-4, which act on the nociceptive neurons ASH and ADL (13, 14). Interactions between GLB-5 and the H-NOX domains of GCY-35 and GCY-36 are essential for rapid adaptation to low or high oxygen levels (15).

**Figure 1:**
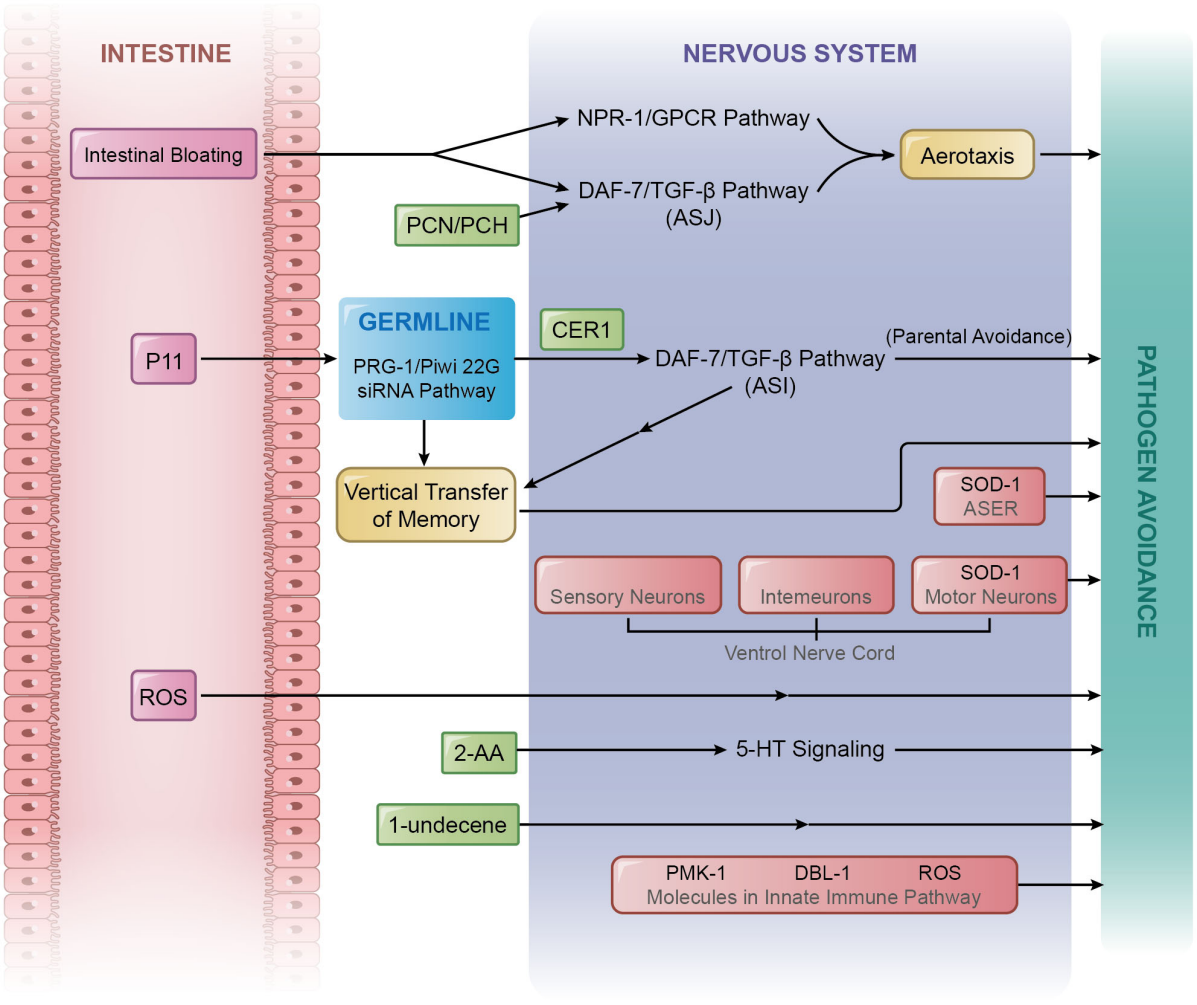
Mechanisms of pathogen avoidance. A variety of comprehensive mechanisms are in place to regulate pathogens avoidance, which include those caused by the alteration of aerotaxis, intestinal bloating, metabolites, transgenerational epigenetic inheritance, and crosstalk with innate immunity.

PA14, consumes oxygen, results in a decrease in the surrounding oxygen level (11, 12), which may attract and subsequently harm *C. elegans*. However, *C. elegans* have developed the capacity to adapt to their preferences through the alteration of aerotaxis to counter the challenge of lower oxygen surroundings, enabling them to avoid PA14 (**Figure 1**).

A critical component of the adaptive response is the DAF-7/TGF-β signalling pathway in ASJ neurons of *C. elegans* (16). Detailed chemical analysis of secondary metabolites of PA14 has identified two chemical components, phenazine-1-carboxamide (PCN) and pyochelin (PCH), as potent stimulators of *daf-7* gene expression as well as the activation of the TGF-β signalling pathway in ASJ neurons. Consequently, the sensation of PCN and PCH is believed to be a crucial step in the alteration of aerotactic behaviour and promotes PA14 avoidance (16).

### 2.2 Intestinal bloating

Intestinal bloating is identified as the expansion of the intestinal lumen (17). Researchers have discovered a correlation between the degree of intestinal bloating and the degree of pathogen avoidance; reduced intestinal bloating led to delayed avoidance, whereas increased bloating enhanced avoidance (17). Further research confirmed that the DAF-7/TGF-β signalling pathway could be stimulated during intestinal bloating and that intestinal bloating does, indeed, contribute to pathogen avoidance triggered by aerotaxis alteration (17, 18). Moreover, intestinal bloating and ASJ neuron detection of PCN and PCH trigger aerotactic changes, initiating pathogen avoidance. A recent study reported that intestinal bloating can stimulate histone H4 Lys8 acetylation in the *C. elegans* germline, which requires the participation of PAR-5, a protein belonging to the 14-3-3 chaperone protein family. This process is pivotal in pathogen avoidance, potentially acting as an intermediary in the signalling pathway between the intestine and neurons (19). The ASJ neurons contribute significantly to aerotactic responses resulting in pathogen avoidance; however, the roles of AWB, AWC, or ADF neurons remain unclear. Future investigations are warranted because of the critical role these neurons play in forming the neural circuitry for pathogen avoidance. In addition to the DAF-7/TGF-β pathway, another neuroendocrine pathway, NPR-1-mediated signalling, is also essential for pathogen avoidance triggered by the alteration of aerotaxis (18, 20, 21). A neuropeptide Y receptor homologue, NPR-1 (22), is expressed in AQR, PQR, and URX sensory neurons. Its ligands are FLP-18 and FLP-21 (23). To function in these neurons, NPR-1 requires TAX-2, TAX-4, and soluble guanylyl cyclase GCY-35 to bind to molecular oxygen (10, 20, 23). Notably, expression of the *npr-1* gene could also be activated by intestinal bloating (17), suggesting a parallel operational mode with the DAF-7/TGF-β pathway after intestinal bloating. The functions of both DAF-7 and NPR-1 in pathogen avoidance necessitate the expression of the transient receptor potential channel vanilloid genes, *osm-9* and *ocr-2* (10, 16). Furthermore, NPR-1 can be inhibited by HECW-1, an E3 ubiquitin ligase, which functions in outer labial lateral (OLL) sensory neurons, leading to the inhibition of avoidance behaviour (24) (**Figure 1**). These intricate networks of sensory inputs and signalling pathways underscore the complexity and adaptability of pathogen avoidance in *C. elegans* in response to environmental stimuli.

### 2.3 Secondary metabolites

Several types of secondary metabolites secreted by PA14 can be regarded as microbial-associated molecular patterns (25, 26). Some of these serve as stimuli for *C. elegans* to avoid pathogens (**Figure 1**). In addition to PCN and PCH, 2-aminoacetophenone (2-AA) initiates pathogen avoidance. 2-AA is a volatile chemical synthesised by PA14. When exposed to 2-AA, 5-HT signalling in neurons is stimulated. Since 5-HT receptors exist in many types of tissues, the information carried by 5-HT spreads throughout multiple parts of *C. elegans*, including the intestine, germline, and head. Such a reaction results in the enhanced expression of the heat shock factor protein HSF-1 (27) that is required for pathogen avoidance (28). After contact with PA14, HSF-1 assists the rapid transcription of the *hsp-70* gene by association with RNA polymerase II (28). The *hsp-70* gene can encode the chaperone heat shock protein HSP-70 (29). Another effective secondary metabolite of PA14 is 1-undecene, an 11‐carbon olefin, which is a *Pseudomonas*-specific volatile (30). It can be sensed by AWB neurons and elicit pathogen avoidance behavioural responses (31).

## 3 Neural circuit of pathogen avoidance

**Figure 2:**
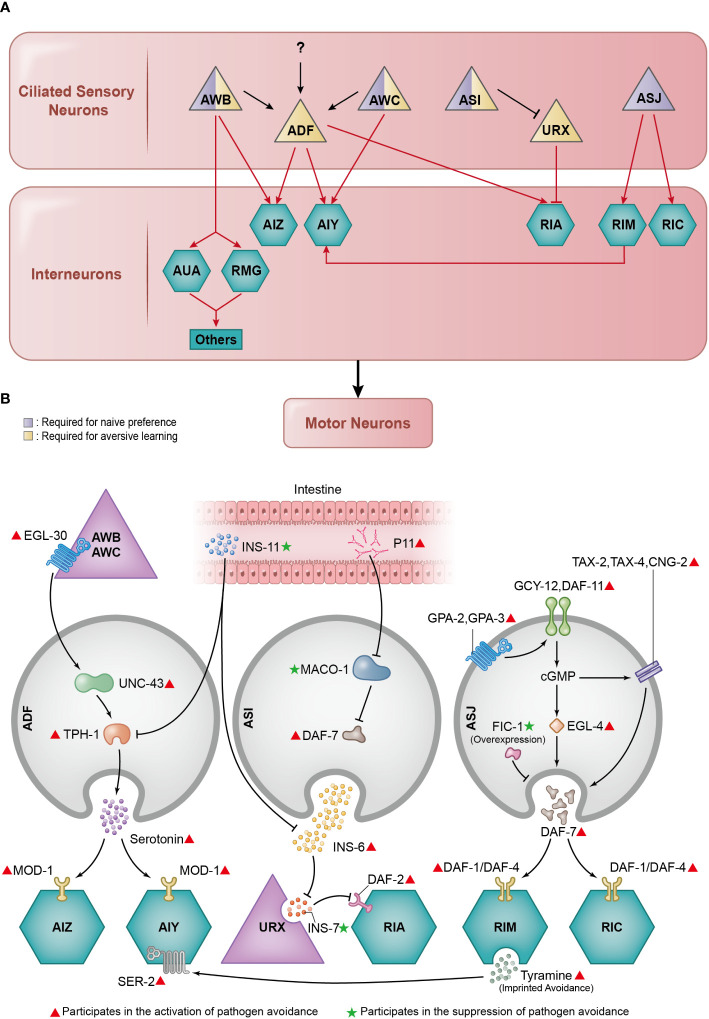
Essential neuronal basis of pathogen avoidance. **(A)** Several neural circuits contribute to the intricate process of pathogen avoidance. In essence, these circuits form a three-layer structure composed of ciliated sensory neurons, interneurons, and motor neurons. The sensory neurons play distinct roles: some are tasked with naïve preference, others with learned avoidance, and a select few handle both. **(B)** The regulation of pathogen avoidance is a collaborative effort between neurotransmitters and neuromodulators. Working in unison, they meticulously calibrate the process of pathogen avoidance.

Neurons serve as information perception and integration centres in *C. elegans*. They manipulate all behaviours in response to varying environmental stimuli. When a threat from PA14 arises, they play an indispensable role in controlling pathogen avoidance behaviour through a series of intricate molecular events (**Figure 2**).

Upon PA14 exposure, *C. elegans* undergoes two phases of pathogen avoidance behaviour: initial attraction and subsequent repulsion. Initially, *C. elegans* is attracted to PA14 via the process of naïve preference, but over time, *C. elegans* avoids PA14 through the mechanism of learned avoidance (9). At least three different interconnected neural circuits participate in this process.

The first is the AWB-AWC sensorimotor circuit, which is responsible for both initial naïve preference and learned avoidance (32). The AWB and AWC neurons are located in the anterior region of *C. elegans* and possess cilia that are partially exposed to the external environment (33). When the surrounding bacteria change from the standard laboratory non-pathogenic bacterium *Escherichia coli* OP50 to the pathogenic bacterium PA14, the intracellular calcium dynamics in AWC are inhibited, whereas those in AWB neurons are stimulated (32).

The second neural circuit is the AWB neuron-mediated learned reflexive aversion circuit, devoted to learned avoidance. After aversive learning, *C. elegans* exhibit aversive reflexes on exposure to PA14. Backward locomotion is necessary during aversive reflection. This is controlled by the AWB neuron-mediated learned reflexive aversion circuit, which goes from sensory neurons AWB to interneurons AUA/RMG, progresses to lower-layer interneurons AVA/AVD/AVE, and ultimately reaches motor neurons VA/DA/AS/VA/DD (34).

The third circuit is the ADF modulatory neural circuit dedicated to learned avoidance, although ADF neurons themselves have a slight effect on naïve preference (32). ADF neurons are located in the anterior region of *C. elegans* and also possess cilia exposed to the environment (33). Upon exposure to PA14, increased serotonin secretion is triggered. The serotonin produced by ADF further signals the serotonin-gated chloride channel MOD-1 in the downstream AIY and AIZ interneurons, inducing learned avoidance through aversive learning (9, 35). Serotonin production in the ADF neurons relies on the rate-limiting enzyme tryptophan hydroxylase-1 (TPH-1), which is upregulated upon pathogen exposure. During this process, Gqα protein EGL-30 in upstream AWB and AWC neurons induces the expression of CaMKII/UNC-43 in ADF neurons, leading to the increased expression of *tph-1* (36). Notably, there is also a down-regulatory mechanism for *tph-1*; exposure to pathogens triggers *ins-11* expression in the intestine via the p38 MAPK signalling pathway and the transcription factor EB-coding gene *hlh-30*. The increased INS-11 levels subsequently reduce the basal expression levels of *tph-1* (37). These three preliminarily identified circuits share multiple common neurons, but how they interact with each other remains to be further investigated.

Serotonin is not only pivotal in aversive learning in *C. elegans* but may also play a similar role in humans. Disgust is hypothesised to function as an adaptive pathogen avoidance mechanism in humans, preventing exposure to parasites, including bacteria (38). This behaviour manifests in two phases: prior disgust, which occurs without contact with the pathogen, and posterior disgust, which occurs after contact (39). The posterior disgust phase often leads to vomiting, a process in which serotonin serves as a critical signalling molecule (39, 40).

While the aforementioned *C. elegans* neural circuits predominantly involve the AWB, AWC, and ADF neurons in pathogen avoidance behaviour, it is crucial to consider the roles of other ciliated sensory neurons located in the anterior region of *C. elegans*, such as the ASI and ASJ neurons (33). Although they do not form clearly specified circuits, as outlined before, ASI and ASJ neurons contribute significantly to pathogen avoidance. The ASI neurons are required for naïve preference (41) and learned avoidance (33). The ASJ neurons are required for naïve preference (16), but their role in learned avoidance has not been definitively established.

The ASI neurons secrete insulin-like neuropeptide INS-6, which suppresses the expression of insulin-like neuropeptide INS-7 in URX sensory neurons. INS-7, in turn, inhibits the insulin receptor DAF-2 in RIA interneurons. Since DAF-2 in the RIA interneurons positively regulates learned pathogen avoidance, the neuronal cascade initiated from the secretion of INS-6 from ASI neurons also positively regulates learned pathogen avoidance (42). The pathway responsible for the activation of *ins-11* described earlier could suppress the expression of *ins-6* in the ASI neurons, contributing to the reduction of aversive learning behaviours (37). On the other hand, a recent study reported that the small RNA P11 (sRNA P11) of PA14 could down-regulate the expression of the *maco-1* gene, an endoplasmic reticulum (ER) membrane protein coding gene, causing activation of the DAF-7/TGF-β pathway in ASI neurons, thereby promoting pathogen avoidance (41).

In ASJ neurons, the G protein-coupled receptors (GPCR) GPA-2 and GPA-3 are activated during pathogen exposure. GPCRs then stimulate the guanylate cyclases DAF-11 and GCY-12 to produce cGMP, which activates the cGMP-dependent kinase EGL-4 and the cyclic nucleotide–gated (CNG) ion channels TAX-2, TAX-4, and CNG-2. Together, these proteins initiate the rapid transcription of the *daf-7* gene (43). The *daf-7* gene encodes neuromodulator DAF-7 that can bind with TGF-β receptor DAF-1/DAF-4 on downstream interneurons RIM and RIC, activating R-SMADs DAF-8/DAF-14 and then suppressing co-SMAD DAF-3, resulting in enhanced pathogen avoidance by modulation of aerotaxis (16). The expression of *daf-7* in the ASJ neurons and its downstream TGF-β pathway could be suppressed by the overexpression of FIC-1 (44), which belongs to the family of AMPylase containing the Fic domain (45, 46). Additionally, ASJ neurons can sense nitric oxide (NO) to initiate pathogen avoidance, a process that requires the CNG channels TAX-2 and TAX-4 and the receptor guanylate cyclase DAF-11. When NO is present or removed, the calcium levels increase in ASJ neurons. This phenomenon is influenced by TRX-1/thioredoxin, a redox-sensing protein (47). The presence of cilia in all the previously described neurons underlines their significance in the sensation of external stimuli (48–50), illustrating the crucial role of ciliary structures in the behavioural pathogen avoidance.

Another noteworthy phase of avoidance is known as imprinted aversive learning. When exposed to PA14 shortly after hatching, *C. elegans* undergo long-term imprinted aversive learning. The formation of this long-term memory involves the sensory neurons AIB and interneurons RIM, whereas the retrieval of memory relies on the sensory neurons AIY and interneurons RIA. Specifically, the signals are transmitted by the neuromodulator tyramine, which is secreted by the RIM and binds to the tyramine receptor SER-2 in AIY. Additionally, imprinted aversive learning requires the participation of serotonin secretion-related genes, including *tph-1* and *mod-1*, glutamine secretion -related genes *eat-4*, *glr-1*, and *glr-3*, as well as the cAMP response element-binding protein-coding homolog *crh-1* (35).

## 4 Epigenetic inheritance of pathogen avoidance

The phenomenon of epigenetic inheritance, in which certain behaviours are transmitted across generations, has been observed and studied in various species. In the case of *C. elegans*, this intriguing phenomenon is evident in the context of pathogen avoidance. When maternal *C. elegans* were exposed to PA14 for 24 h, enhanced naïve preference and learned avoidance response to PA14 were observed in at least four subsequent generations (51).

In many animals, the epigenetic inheritance of behaviour across generations requires multiple small noncoding RNA pathways (52, 53). Similarly, in *C. elegans*, the epigenetic inheritance of pathogen avoidance behaviour critically relies on small noncoding RNA pathways, specifically the PRG-1/Piwi 22G siRNA pathway. Notably, the role of DAF-7/TGF-β signalling within the ASI neurons is crucial to this process (51). Researchers revealed that PA14 exposure leads to the generation of abundant PIWI-interacting RNAs in maternal *C. elegans*. Subsequently, a sequence involving the Piwi argonaute protein PRG-1 (54), RNA-dependent-RNA-polymerase RRF-1 (55), and RNaseD homologue MUT-7 (56) generates secondary endo-siRNAs (22G RNAs) using PIWI-interacting RNAs as sources (51). These siRNAs then translocate to the nucleus and guide the expression of histone methyltransferase SET-25 (57) and histone receptor HPL-2 (58), leading to chromatin modifications and the epigenetic inheritance of pathogen avoidance in subsequent generations (51). Intriguingly, parental PA14 exposure duration influences progeny behaviour; a short four-hour exposure results in progeny preference for PA14, whereas an eight-hour exposure switches the response to avoidance. The Piwi argonaute protein PRG-2 (59) is involved in this modulation (60).

Further research revealed a more intricate mechanism underlying epigenetic inheritance via the PRG-1/Piwi 22G siRNA pathway (41). This process is triggered by a specific noncoding RNA, sRNA P11, which is exclusive to pathogenic PA14. It induces learned avoidance in the maternal *C. elegans* and their progeny. Elements in the RNA interference pathway are key to maternal learned avoidance: Double-stranded RNA (dsRNA) transporter SID-2 (61) and dsRNA endoribonuclease Dicer DCR-1 (62) are required for response to PA14 sRNA; AGO3 homolog RDE-1 (63), RNA interference-defective protein RDE-2 (64), RDE-4 (65), and MUT-7 (56) are required for response to sRNAs of both PA14 and OP50 (41); dsRNA transporter SID-1 (66) is required for response to sRNAs of both PA14 and OP50, as well as naïve preference. After the ingestion of sRNA P11, the PRG-1/Piwi 22G siRNA pathway in the germline is activated. It is essential for the transgenerational epigenetic inheritance of pathogen avoidance in progenies induced by P11. Several elements in the PRG-1/Piwi 22G siRNA pathway are essential: Piwi argonaute protein PRG-1, RNA-dependent RNA polymerases RRF-1 (67) and, RRF-3 (68), and heterochromatin regulator HPL-2 (41). In germlines, the virus-like particle transposon Cer1 loads RNAs and carries signals from germlines to the ASI neurons (69–71). This further downregulates the expression of the ER membrane protein MACO-1, which in turn up-regulates DAF-7 in the ASI neurons, resulting in maternal learned avoidance and epigenetic inheritance of pathogen avoidance in progenies (41). Besides sRNA, intestinal bloating could also induce the transgenerational inheritance of pathogen avoidance, which is accompanied by chromatin modification involving H4 Lys8 acetylation in germlines (19) (**Figure 2**).

## 5 Signalling crosstalk between pathogen avoidance and innate immunity

The pathogen avoidance behaviour of *C. elegans* coordinates their escape from pathogens to limit infection. In addition, *C. elegans* also initiate certain mechanisms of the innate immune system to eliminate bacteria to prevent pathogen invasion. Although the modes of activation and mechanisms of pathogen avoidance and innate immunity are quite different, several studies identified evidence for signalling crosstalk between these defence strategies.

Pathogen avoidance can be induced by several classical factors in the innate immune system. One key factor are reactive oxygen species (ROS). Bacteria ingested by *C. elegans* are usually transferred to the intestine by movement of the pharynx. Ingested PA14 accumulates in the intestine, resulting in ROS production (72, 73). ROS, which include hydroxyl radicals, superoxide anions, and hydrogen peroxide, are generated from oxygen reduction. While excessive ROS can be toxic to cells (74–77), they also serve a protective function by killing invading bacteria and fungi (72, 78), suggesting dual roles in innate immunity. Recent studies demonstrated that ROS also affect pathogen avoidance (**Figure 1**).

Exposure to PA14 triggers ROS generation in the *C. elegans* intestine, promoting the expression of the antioxidant enzyme superoxide dismutase-1 (SOD-1) in intestinal ASER neurons (79). SOD-1 protects cells by converting superoxide into less toxic oxygen or hydrogen peroxide (80, 81). The induction of SOD-1 in ASER neurons results in the inhibition of the pathogen avoidance at first. Subsequently, SOD-1 levels return to normal when *C. elegans* show a tendency toward pathogen avoidance. Although SOD-1 is expressed in the cell body, dendrites, and cilia of ASER, those expressed in the cilia are essential for pathogen avoidance (79). Furthermore, guanylyl cyclases GCY-22 and GCY-5 mediate SOD-1 induction in ASER neurons (82), while NPR-1 suppresses SOD-1 expression (83). Interestingly, another antioxidant enzyme, SOD-5 (81), also suppresses pathogen avoidance (83).

In addition to the ASER neurons, SOD-1 is also expressed in the ventral nerve cords, specifically in the cholinergic motor neurons. The ventral nerve cords are composed of sensory neurons, interneurons, and motor neurons. The AMPA-type ionotropic glutamate receptor GLR-1 in interneurons (84) can suppress SOD-1 expression upon pathogen exposure (84). Lack of GLR-1 results in enhanced SOD-1 expression and enhanced pathogen avoidance, indicating that GLR-1 negatively regulates pathogen avoidance in a non-cell-autonomous manner. As GLR-1 is a glutamate receptor, glutamatergic sensory neurons upstream of the interneurons where GLR-1 is expressed may also be involved in pathogen avoidance. Indeed, lack of the vesicular glutamate transporter EAT-4 (85) in glutamatergic sensory neurons could regulate pathogen avoidance. Specifically, rather than promoting or suppressing avoidance, the loss of EAT-4 causes *C. elegans* to lose the ability to distinguish between the pathogenic bacterium PA14 and the non-pathogenic bacterium *E. coli* OP50 (86).

In the intestine, PA14 induces ROS production in intestinal cells. ROS increases the formation of oxidised glutathione or GSSG. GSSG can be transported into the pseudocoelomic cavity by the gut efflux pump MRP-1, an ATP-binding cassette transporter located in the basolateral membrane of intestinal cells. Subsequently, GSSG triggers pathogen avoidance. This process requires the NMDA class glutamate receptor-1 NMR-1 in downstream neurons. Researchers verified this by demonstrating that extracellular GSSG supplementation activated pathogen avoidance, while loss of either MRP-1 or NMR-1 resulted in the inhibition of aversive learning (87). Another study found out that NMR-1 in the RIM neurons decreases INX-4 abundance via UNC-43 to diminish the strength of the gap junction in the RIM-circuit, which contributes to the pathogen avoidance (88). Given that ROS can impair intestinal cell functions, potentially leading to intestinal damage and subsequent bloating, it would be worthwhile to determine whether there are connections between the avoidance caused by intestinal bloating and ROS production.

In addition to ROS, several critical proteins within the classical immune pathway play a role in activating pathogen avoidance behaviours. One key protein is DBL-1, a ligand that initiates the DBL-1/TGF-β innate immune signalling pathway (89, 90). Research has demonstrated that DBL-1 is essential for aversive learning (91). Researchers found that the exposure to PA14 decreases the calcium response in AVA interneurons. This diminished AVA neuronal activity triggers the secretion of DBL-1, which then binds to the type I TGF-β receptor SMA-6 in the hypodermis. Subsequent TGF-β signalling facilitates aversive learning, enabling the organism to actively avoid PA14 (91). Another crucial factor is PMK-1, a core molecule in the p38 MAPK pathway (92), which acts as a negative regulator of pathogen avoidance (93). A deficiency of innate immunity caused by the loss of PMK-1 can stimulate avoidance behaviour in *C. elegans*. Within the OLL neurons, this loss suppresses the expression of the *hecw-1* gene, which encodes an E3 ubiquitin ligase. Suppression of this ligase exerts a non-cell-autonomous effect, increasing NPR-1 expression in the RMG neurons and ultimately activating the pathogen avoidance response (93).

Multiple factors involved in pathogen avoidance can induce innate immunity. NPR-1 can positively control innate immunity by inhibiting the activity of neurons AQR, PQR, and URX, which transfer neuroendocrine signals through pseudocoelomic body fluid to non-neural tissues and negatively regulate the innate immunity (94). Likewise, intestinal bloating upregulates multiple innate immune genes, such as *clec-60, lys-3, lys-4, ilys-3, cpr-2*, and *F53A9.8* (17). Furthermore, the secondary metabolite PCN can bind to the nuclear hormone receptor NHR-86/HNF4 in intestinal epithelial cells. This binding triggers the expression of multiple immune genes, initiating a transcriptional program that enhances innate immunity (95).

## 6 Pathogen avoidance of non-*Pseudomonas* bacteria

While PA14 is the most studied pathogen for avoidance behaviours, there are also extensive studies on the mechanisms of pathogen avoidance of non-*Pseudomonas* bacteria species, such as *Bacillus thuringiensis* (BT) and *Enterococcus faecalis* (EF).

BT, a gram-positive, rod-shaped aerobic bacterium, produces Cry toxin, a δ-endotoxin that can undergo oligomerisation (96, 97). Cry toxins are widely used as a bioinsecticide (97). *C. elegans* tends to avoid BT with an observed capacity to differentiate between strains of varying pathogenicity, preferentially evading more virulent strains. *C. elegans* with *npr-1(ur89)* mutations exhibit stronger avoidance behaviour. The RNA-seq profile of *npr-1(ur89)* mutants suggests the potential involvement of Ebox transcription factors, oxidative stress genes, p38 MAPK signalling, C-type lectins, and insulin-like signalling in the avoidance of BT (98). Two studies confirmed the participation of C-type lectins and insulin-like signalling in pathogen avoidance. One study revealed that the mutation of the C-type lectin gene *C54G4.4* exhibited increased BT avoidance (99). Another study demonstrated that two main components of insulin-like signalling, the receptor DAF-2 and the transcription factor DAF-16, are critical for BT avoidance (100). Notably, one of the Cry toxins secreted by BT, Cry6Aa2, induces avoidance in the absence of BT (101).

EF is a gram-positive, spherical or ovoid-shaped and facultative anaerobic bacterium (102, 103). *C. elegans* initially exhibit avoidance of the EF lawn, followed by periodic returns, forming a cyclic behavioural pattern with peak avoidance observed approximately four hours post-exposure (104). Exposure to EF causes intestinal bloating, which results in pathogen avoidance mediated by AWB and AWC neurons. Avoidance is also contingent on the presence of the NPR-1, TAX-2, and TAX-4 proteins in conjunction with ASE neurons. Additionally, GON-2 and GTL-2, two transient receptor potential melastatin channels, have been identified as mediators of this avoidance response (104). The sensorimotor circuit for learned avoidance of EF is initiated by the AWB sensory neurons, proceeding through the interneurons AUA/RMG, extending to the lower layer interneurons AVA/AVD/AVE, and ultimately reaching the motor neurons VA/DA/AS/VA/DD (34).

## 7 Concluding remarks

From the phenotypic to the molecular level, studies on pathogen avoidance in *C. elegans* have come a long way. The interpretation of pathogen avoidance has enriched our knowledge of these sophisticated behaviours. In this paper, we reviewed the stimuli that trigger pathogen avoidance, including alterations in aerotaxis, intestinal bloating, and metabolites. Furthermore, we summarised the neural circuits in pathogen avoidance, transgenerational epigenetic inheritance of pathogen avoidance, signalling crosstalk between pathogen avoidance and innate immunity, and *C. elegans* avoidance of non-pathogenic bacteria. Diverse pathogen avoidance mechanisms provide comprehensive and adaptive protection, enabling multiple reactions to occur in complex environments. Collectively, these mechanisms contribute to the evolutionary advantages of *C. elegans*. Future studies could investigate how each mechanism contributes to this complex behaviour. It would also be valuable to investigate the interactions that may occur among pathogen avoidance mechanisms. Given the evolutionary conservation between *C. elegans* and humans, these studies may shed light on our understanding of gut neural signalling in humans, thus providing insight into potential therapies for treating bacterial infections.

## Author contributions

ML: Writing – original draft, Writing – review & editing. YT: Writing – original draft, Writing – review & editing. HT: Writing – original draft, Writing – review & editing. WT: Writing – original draft, Writing – review & editing.

## Glossary

**Table:**

Names	Description
C54G4.4	Sushi, von Willebrand factor type A, EGF and pentraxin domain-containing protein 1
*clec-60*	VWFA domain-containing protein
CNG-2	Cyclic nucleotide-binding domain-containing protein
*cpr-2*	Peptidase C1A papain C-terminal domain-containing protein
*creb-1/crh-1*	CREB homolog *crh-1*
DAF-1	Cell surface receptor *daf-1*
DAF-2	Insulin-like receptor subunit beta; Protein kinase domain-containing protein;receptor protein-tyrosine kinase
DAF-3	Smad protein *daf-3*
DAF-4	Cell surface receptor *daf-4*; Serine/threonine-protein kinase receptor; receptor protein serine/threonine kinase
DAF-7	Dauer larva development regulatory growth factor *daf-7*
DAF-8	Smad protein *daf-8*
DAF-11	Receptor-type guanylate cyclase *daf-11*; guanylate cyclase
DAF-14	Smad-related protein *daf-14*
DBL-1	Predicted to enable BMP receptor binding activity; cytokine activity; and protein serine/threonine kinase activator activity.
DCR-1	Death-promoting deoxyribonuclease
EAT-4	Major facilitator superfamily (MFS) profile domain-containing protein; putative vesicular glutamate transporter
EGL-4	cGMP-dependent protein kinase; cGMP-dependent protein kinase *egl-4*
EGL-30	G protein subunit alpha q; Guanine nucleotide-binding protein G(Q) subunit alpha
F53A9.8	Zinc transporter Slc39a7
FIC-1	Protein adenylyltransferase *fic-1*
FLP-18	SVPGVLRF-amide 3
FLP-21	GLGPRPLRF-amide
GCY-5	Receptor-type guanylate cyclase *gcy-5*
GCY-12	Receptor-type guanylate cyclase *gcy-12*
GCY-22	Receptor-type guanylate cyclase *gcy-22*
GCY-35	Guanylate cyclase domain-containing protein; Soluble guanylate cyclase gcy-35
GCY-36	Soluble guanylate cyclase *gcy-36*
GLB-5	Globin family profile domain-containing protein
*glr-1*	Glutamate receptor 1
*glr-3*	Glutamate receptor ionotropic, kainate *glr-3*
GON-2	LSDAT_euk domain-containing protein; Transient receptor potential channel
GPA-2	Guanine nucleotide-binding protein alpha-2 subunit
GPA-3	Guanine nucleotide-binding protein alpha-3 subunit
GTL-2	LSDAT_euk domain-containing protein
*hecw-1*	E3 ubiquitin-protein ligase *hecw-1*
*hlh-30*	Helix-loop-helix protein 30
HPL-2	Chromobox protein homolog *hpl-2*
HSF-1	Heat shock transcription factor *hsf-1*
*hsp-70*	Heat shock protein 70
*ilys-3*	Invertebrate-type lysozyme 3
INS-6	putative insulin-like peptide beta-type 5
INS-7	Insulin-like peptide 7; putative insulin-like peptide beta-type 4
INS-11	B-chain-like peptide
INX-4	Innexin
*lys-3*	Lysozyme-like protein 3
*lys-4*	Lysozyme
MACO-1	Macoilin, an ER membrane protein
MOD-1	Serotonin-gated chloride channel *mod-1*
MRP-1	Multidrug resistance-associated protein 1
MUT-7	Exonuclease *mut-7*
NHR-86	Nuclear hormone receptor family member *nhr-86*
NMR-1	LITAF domain-containing protein
NPR-1	Neuropeptide receptor *npr-1*
OCR-2	Ion transport domain-containing
ODR-4	Odorant response abnormal protein 4
OSM-9	Ion transport domain-containing
PAR-5	14-3-3 domain-containing protein; 14-3-3-like protein 1
PMK-1	Mitogen-activated protein kinase *pmk-1*
PRG-1	Piwi-like protein 1
PRG-2	Piwi (fruitfly) Related Gene
RDE-1	Piwi domain-containing protein
RDE-2	CABIT domain-containing protein; SH2 domain-containing protein
RDE-4	DRBM domain-containing protein
RRF-1	RNA-directed RNA polymerase
RRF-3	RNA-directed RNA polymerase
SER-2	G-protein coupled receptors family 1 profile domain-containing protein; Tyramine receptor *Ser-2*
SET-25	Histone-lysine N-methyltransferase *set-25*
SID-1	Systemic RNA interference defective protein 1
SID-2	Systemic RNA interference defective protein 2
SKN-1	BZIP domain-containing protein; Protein skinhead-1
SMA-6	Serine/threonine-protein kinase receptor *sma-6*
SOD-1	Superoxide dismutase [Cu-Zn]
SOD-5	Superoxide dismutase [Cu-Zn]
TAX-2	Cyclic nucleotide-binding domain-containing protein
TAX-4	Cyclic nucleotide-gated cation channel
TPH-1	Biopterin-dependent aromatic amino acid hydroxylase family profile domain-containing protein
TRX-1	Thioredoxin-1
UNC-43	Calcium/calmodulin-dependent protein kinase II association-domain domain-containing protein; Calcium/calmodulin-dependent protein kinase type II; calcium/calmodulin-dependent protein kinase
ADF	Amphid Dual Ciliated Ending F
ADL	Amphid Dual Ciliated Ending L
AIB	Anterior Interneuron B
AIY	Anterior Interneuron Y
AIZ	Anterior Interneuron Z
AQR	Anterior, Q-cell Derived Receptor
ASER	Amphid Single Cilium E Right
ASH	Amphid Single Cilium H
ASI	Amphid Single Cilium I
ASJ	Amphid Single Cilium J
AS	A-type Short Motor Neuron
AUA	Amphid-associated Unknown Receptor A
AVA	Anterior Ventral Process A
AVD	Anterior Ventral Process D
AVE	Anterior Ventral Process E
AWB	Amphid Wing Neuron B
AWC	Amphid Wing Neuron C
DA	Dorsal A-type Motor Neuron
DD	Dorsal D-type Motor Neuron
OLL	Outer Labial Lateral Dendrite
PQR	Posterior Q-cell Derived Receptor
RIA	Ring Interneuron A
RIC	Ring Interneuron C
RIM	Ring Interneuron M
RMG	Ring Motor Neuron G
URX	Unknown Receptor, not Ciliated X
VA	Ventral A-type Motor Neuron
